# Genetic isolation and morphological divergence mediated by high-energy rapids in two cichlid genera from the lower Congo rapids

**DOI:** 10.1186/1471-2148-10-149

**Published:** 2010-05-19

**Authors:** Jeffrey A Markert, Robert C Schelly, Melanie LJ Stiassny

**Affiliations:** 1 Department of Ichthyology, American Museum of Natural History, Central Park West at 79th Street, New York, NY 10024-5192, USA

## Abstract

**Background:**

It is hypothesized that one of the mechanisms promoting diversification in cichlid fishes in the African Great Lakes has been the well-documented pattern of philopatry along shoreline habitats leading to high levels of genetic isolation among populations. However lake habitats are not the only centers of cichlid biodiversity - certain African rivers also contain large numbers of narrowly endemic species. Patterns of isolation and divergence in these systems have tended to be overlooked and are not well understood.

**Results:**

We examined genetic and morphological divergence among populations of two narrowly endemic cichlid species, *Teleogramma depressum *and *Lamprologus tigripictilis*, from a 100 km stretch of the lower Congo River using both nDNA microsatellites and mtDNA markers along with coordinate-based morphological techniques. In *L. tigripictilis*, the strongest genetic break was concordant with measurable phenotypic divergence but no morphological disjunction was detected for *T. depressum *despite significant differentiation at mtDNA and nDNA microsatellite markers.

**Conclusions:**

The genetic markers revealed patterns of philopatry and estimates of genetic isolation that are among the highest reported for any African cichlid species over a comparable geographic scale. We hypothesize that the high levels of philopatry observed are generated and maintained by the extreme hydrology of the lower Congo River.

## Background

Population boundaries are largely determined by local environment, the strength of barriers to migration, and the organism's inherent dispersal abilities. The interaction among these and genetic factors determines the potential for evolutionary divergence, and is at the heart of our understanding of ecological adaptation and ongoing speciation processes. When individuals can easily cross barriers between suitable habitat patches, gene flow acts to homogenize most neutral genetic diversity and may swamp the effects of any local phenotypic selection whereas low levels of migration may facilitate ecological divergence, even when selection is modest. Evolutionary divergence through fine-scale geographic isolation is believed to be one of the main mechanisms promoting speciation in the cichlid radiations of the African Great Lakes [1-3]. Many lake species are extreme local endemics, with distributions limited to small patches of ecologically distinct habitat. Many narrowly endemic cichlid species are confined to rocky sections along the shores of the lakes separated by stretches of sandy, muddy or weedy shoreline or by deep channels [4]. In several cases, molecular markers have shown that small patches of sand or mud [5,6], intervening deep channels [7], and the mouths of streams and rivers [8] are all noteworthy barriers to migration, and some species even show a pattern of isolation by distance along continuous habitat [9]. Cichlid philopatry in the African Great Lakes may augment the effects of disruptive selection on phenotypic traits associated with mate choice and trophic adaptations believed to be important in species divergence.

Comparatively little is known about the population structure and extent of cichlid philopatry in river systems. In South American cichlids, Ready et al. [10,11], have detected only modest levels of genetic divergence along the Amazon River and a strong pattern of divergence between drainages in the eastern Amazon basin in two different cichlid genera but on a much larger geographic scale than has been observed in African lake cichlids. Similarly, only a few published studies of African river cichlids have focused on broad scale phylogeographic patterns. For example, Wu's study of the habitat generalist *Astatoreochromis alluaudi *throughout the Lake Victoria Basin found little population differentiation[12], whereas Hassanan and Gilbey [13] detected only modest levels of differentiation among *Oreochromis niloticus *populations separated by more than 100 km. In the haplochromine genus *Pseudocrenilabrus*, Katongo et al. [14] detected mtDNA haplotype and morphological differences on broad geographic scales suggesting a pattern consistent with geographic speciation.

The proximate causes and adaptive significance of cichlid philopatry have long been the subject of much speculation. In the African Great Lakes, proposed advantages that may indirectly promote philopatry include predator avoidance [15], microallopatric divergence [15], and reduced mating success for immigrant males who may lack the locally preferred phenotype [8]. The pattern could also be a byproduct of extended brood care or male territoriality (e.g. [16,17]).

While considerably less rich than the African Great Lakes, the shoreline habitats of the Lower Congo River (LCR) nonetheless harbor a large number of cichlid species, and to date 21 narrowly endemic species have been reported from this short stretch of river. One of these, *Steatocranus mpozoensis*, is known also to occur in the Mpozo River, a large leftbank tributary of the LCR. *Thoracochromis demeusii *occurs predominantly in the main channel but is also often found in lower reaches of numerous affluent streams, while the remaining LCR endemics are found exclusively in marginal habitats along the river's main channel. Interestingly there are also parallels in habitat type between the Great Lakes and the LCR system. The eddies along many sections of the LCR shoreline encompass patches of rocky, sandy, muddy, and weedy substrates that resemble the mosaics of habitat types described in the African Great Lakes [4]. Unlike the lacustrine setting, in large rivers, the presence of strong currents might also influence both the distribution of and connectivity among cichlid populations. In particular, the complex hydrology of the LCR provides the potential for additional barriers to inter-population migration that are absent in lacustrine settings.

The Congo River drains approximately 3.8 million km^2 ^section of equatorial Africa containing the largest continuous rainforest on the continent and a water volume that is five times larger than that of the Mississippi River. Near the twin cities of Kinshasa (Democratic Republic of Congo) and Brazzaville (Republic of Congo) the Congo River spills over a rocky sill that forms the eastern boundary of the LCR and plunges through a narrow gorge descending some 280 m in its 350 km journey to the sea [18]. The river gorge itself contains many width expansions and contractions and can be extraordinarily deep--recent *in situ *depth measurements record the channel to be more than 200 m in some areas [19] forming the deepest river trenches and canyons in the world. This, combined with the high annual discharge, steep elevational incline, fluctuating channel width and complex channel topology creates an extremely energetic flow regime with downstream water velocities in excess of 600 cm/s and with *vertical *water velocities of up to 75 cm/second in some sections [20].

The extreme hydrological conditions with persistent, high in-stream water velocities in the LCR are expected to hinder cross-channel passage and isolate small, epibenthic cichlid populations to shorelines. Furthermore, because cichlids have physoclistous gas bladders, adult fish have limited tolerance for rapid change in water pressure [21], suggesting that few individuals would survive a rapid upward transport from depth through entrainment in dynamic vertically upwelling waters. If these complex and powerful river currents do reduce gene flow this would then facilitate subsequent phenotypic divergence in isolation through natural selection, sexual selection, or genetic drift.

**Figure 1:**
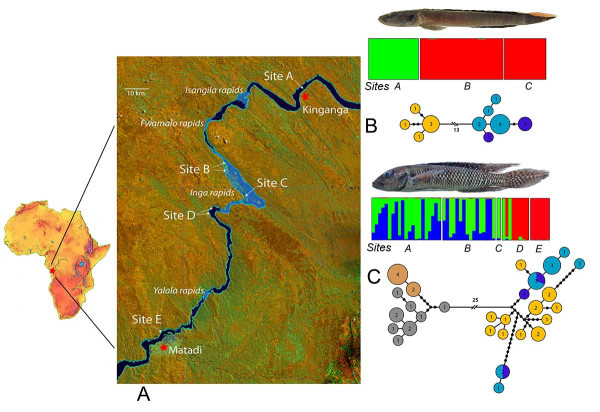
**Patterns of genetic divergence along a 100 km stretch of the Lower Congo River**. Collection sites and major features are shown on the map (A). Assignment test results and mtDNA haplotype networks are shown for T. depressum (B), and for L. tigripictilis (C) populations. Circle size and numbers in center of haplotype networks represent the number of identical haplotypes. Number of substitutions between haplotypes represented by black dots when greater than one, and by a broken line with a number for the longest branches. Collection sites in haplotype networks represented by: yellow = Site A, blue = Site B, purple = Site C, grey = Site D, and tan = Site E.

Here we report the first detailed estimates of interpopulational connectivity and phenotypic divergence among cichlids in a high energy African river. We selected two locally endemic species with distinct ecologies from a 100 km stretch of the LCR (Figure 1-A). *Teleogramma depressum*, is a goby-like, benthic species that is confined to rocky, shoreline habitats and which guard fixed territories (Pers. Obs.). *Teleogramma depressum *appears to be restricted in its geographical distribution to a region approximately matching the present study reach. *Lamprologus tigripictilis *is a habitat generalist found over riffles and rocky habitats as well as over less complex substrates where sand or mud predominate. They have even been occasionally collected in flooded grasses, and are considerably less territorial than *T. depressum *(Pers. Obs.). The known distributional range of *L. tigripictilis *is considerably larger than that of *T. depressum*, and extends beyond the study reach from the region of Mbelo some 100 km downstream from Pool Malebo to Boma about 45 km downstream of Site E near Matadi.

## Results

### Morphological Divergence

**Figure 2:**
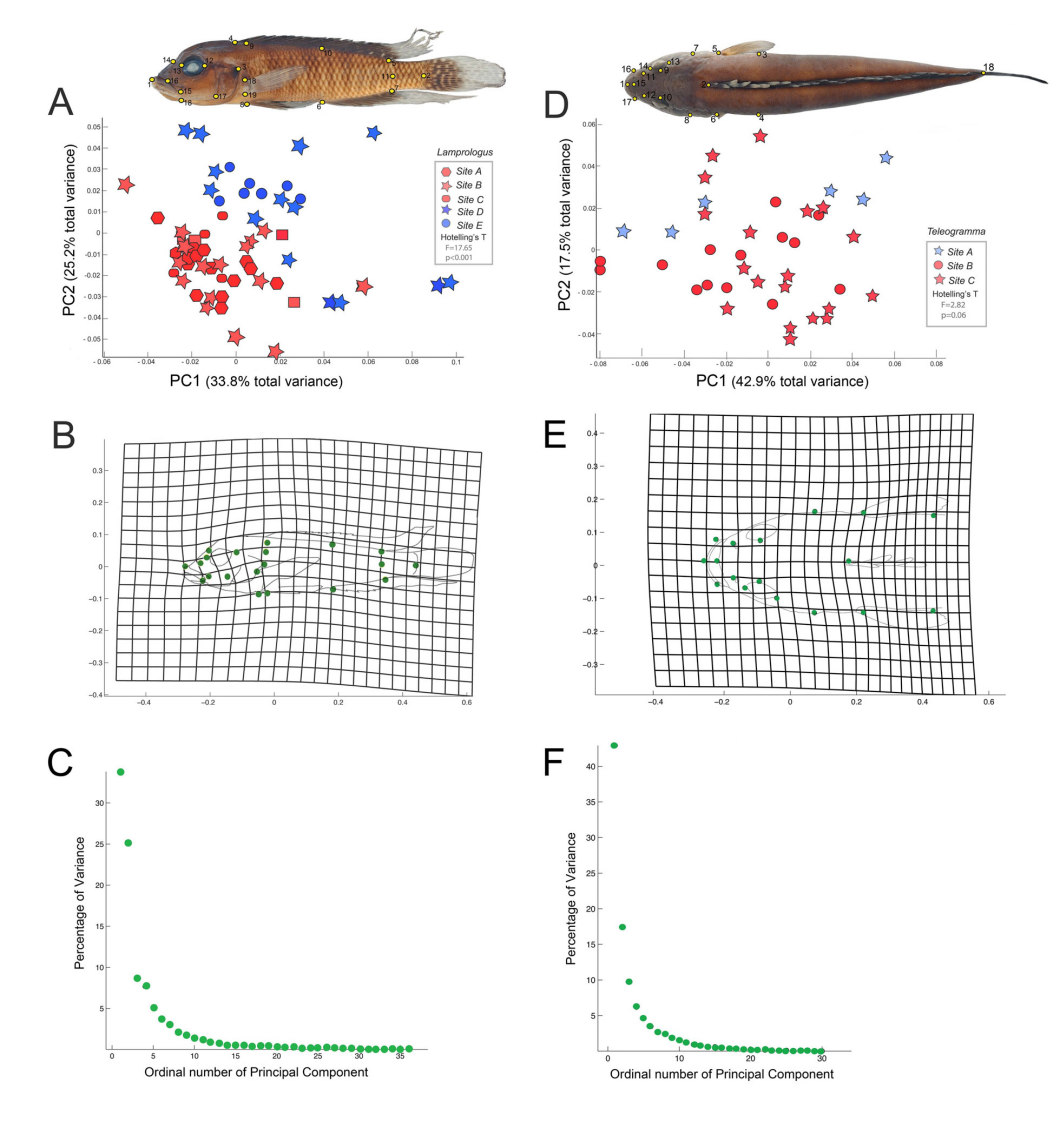
**Principal Components Analysis of body shape variance. ***Lamprologus tigripictilis*. (A) PC1 accounts for 33.8% and PC2 for 25.2% of total shape variance, (B) Deformation implied by PC1 scores using Procrustes superposition, (C) Scree plot of percentage of variance explained by each Principal Component as a % of total variance. *Teleogramma depressum *(D) PC1 accounts for 42.9% and PC2 for 17.5% of total shape variance, (B) Deformation implied by PC1 scores using Procrustes superposition, (C) Scree plot of percentage of variance explained by each Principal Component as a % of total variance.

The results of the coordinate-based morphometric analyses are graphically represented in Figure 2. For *L. tigripictilis*, PCA (Figure 2-A, PC1 accounting for 33.8% and PC2 accounting for 25.2% of total variance, see also Figure 2-C) indicates phenotypic differentiation between individuals from the three upstream sites (Sites A, B, and C) and those of the two downstream sites (Sites D and E). Hotelling's T^2 ^indicates these differences are significant (F = 17.65, df = 40, 23, p < 0.001). A grid display of deformations implied by PC 1 scores using Procrustes superposition highlights regions of shape variation concentrated on mouth size, paired fin placement, and body depth (Figure 2-B). In contrast, PCA results for *T. depressum *(Figure 2-D, PC1 accounting for 42.9% and PC2 accounting for 17.5% of total variance, see also Figure 2-F) revealed no clear relationship between shape variation and geographic location. Deformation implied by PC1 scores using Procrustes superposition (Figure 2-E) indicates little variation among landmark configurations, and no significant differentiation between populations from Sites A and B-C were detected (Hotelling's T^2 ^F = 2.82, df = 34, 8, p = 0.06).

### Genetic Diversity

Sample sizes and genetic diversity summary statistics for the nuclear loci can be found in Table 1. In *T. depressum*, the observed 10 locus mean heterozygosity ranged from 0.36 in the sample from Site C to 0.42 at Sites A and B. All loci were in Hardy-Weinberg equilibrium after using a Bonferroni correction for multiple comparisons, however one locus deviated significantly from Hardy-Weinberg expectations at the p=0.05 level in samples from Sites B and C. Population samples at Sites 2 and 3 were both fixed for single alleles at Locus TmoM25 and at Locus GM-125. *L. tigripictilis *populations showed a clear trend of decreasing heterozygosity from upstream to downstream, with H_obs _ranging from 0.62 at site A to 0.47 at site E., and all loci were in Hardy-Weinberg equilibrium. Within site variation for mtDNA markers is shown in Table 2. No clear upstream - downstream trend in mtDNA diversity was detected.

**Table 1:** Collection localities, sample sizes, and microsatellite summary data.

			*Teleogramma depressum*					*Lamprologus tigripictilis*				
Site	Coordinates	Distance from A	N	Na	**H**_**obs**_	**f**_**st**_	*Nm*	N	Na	**H**_**obs**_	**f**_**st**_	*Nm*
**A**	5° 17.20' S, 13° 44.76' E	0	6	3.3	0.42			21	13	0.62		
						0.231**(0.595)	0				0.013 (0.052)*	3
**B**	5° 27.31' S, 13° 35.39' E	43	10	4.2	0.42			16	10	0.58		
						0	1					
**C**	5° 31.14' S, 13° 37.86' E	51	5	3.5	0.36			1	-	-	0.216 (0.939)**	0.9
						-						
**D**	5° 33.37' S, 13° 33.22' E	62	-	-	-			8	6.4	0.56		
						-					0.132 (0.364)*	0.9
**E**	5° 48.11' S, 13° 27.43' E	100	-	-	-			7	3.5	0.47		

"Distance from A" shows dispersal distances (km) within the river relative to Site A. N = sample size, Na = number of alleles observed per locus, H_obs _= observed heterozygosity. *Nm *= the estimated number of migrants estimated using the private alleles method. Traditional (and standardized) ϕ_st _values are shown between adjacent populations (* p < 0.05, ** p < 0.001).

**Table 2:** Mean number of mtDNA substitutions within collection sites and among collection sites.

	Within Sites		Among Sites				
	
	*Teleogramma*	*Lamprologus*	Site A	Site B	Site C	Site D	Site E
**Site A**	2.00	6.48	-	10.02	-	31.07	34.64
**Site B**	2.67	7.75	17.33	-	-	31.81	35.24
**Site C**	1.25	-	15.88	2.54	-	-	-
**Site D**	-	8.36	-	-	-	-	10.30
**Site E**	-	0.53	-	-	-	-	-

Values for *L. tigripictilis *are shown above the diagonal, those for *T. depressum *are shown below.

The Bottleneck program detected no evidence of a recent bottleneck in any of the populations in either species (Wilcoxan 1 tailed test of H deficiency: p = 0.78 for *L. tigripictilis*, p = 0.24 for *T. depressum*).

### Genetic Divergence

Both mitochondrial and nuclear loci supported the presence of two partially isolated genetic groups for both study species. In *T. depressum *mtDNA revealed two distinct sets of haplotypes that differed by 13 substitutions between the samples from Site A relative to the samples from Sites B and C. Individuals in populations A and B differed by an average of 17 mtDNA base substitutions. Similarly, our analysis of nuclear loci using the assignment test framework found the strongest support for two genetic clusters (Site A) vs. (Sites B & C), (Figure 1(B)). These groups were strongly differentiated, with a microsatellite based ϕ_st _of 0.23 (p < 0.01) (Table 1). Sites B and C were not statistically distinguishable from each other within *T. depressum*.

	The pattern of genetic differentiation was more complex among *L. tigripictilis *populations. The best-supported assignment test models for *L. tigripictilis *had three distinct clusters (Figure 1C). Most individuals collected from sites D and E were united in a single cluster. The ϕ_st _estimate between samples from these two sites is 0.132 and is significantly different from zero (p = 0.004). Individuals from Sites A and B belonged to two distinct clusters, however these clusters were distributed between two collection localities and the probabilities of assigning individuals to one or the other cluster were often low. In an attempt to 'force' these individuals to assign to one cluster or another, a follow up Structure analysis was performed which initialized on the collection localities and which did not allow admixture. However the results from this subsequent analysis were similar to the initial analysis (results not shown). The estimated ϕ_st _of 0.013 between *L. tigripictilis *samples from Sites A and B is modest but statistically significant (p = 0.035). The most dramatic difference in the data set was observed between Site B and Site D, with a ϕ_st _estimate of 0.216 (p = 0.001). The structure results assigned individuals from Sites D and E to a single genetic cluster. The ϕ_st _estimate between samples from these two sites is 0.132 and it is significantly different from zero (p = 0.004). A similar pattern was reflected in the mtDNA data set, with an average of 31 substitutions between individuals at Sites B and D, and two distinct haplotype clusters separated by 25 substitutions (Figure 1-C and Table 2).

Isolation by distance analysis could not be conducted on the *T. depressum *data set because only three geographic localities were successfully sampled. The data were not consistent with a pattern of isolation by distance among *L. tigripictilis *populations (r = 0.48, p = 0.12 for untransformed data). A high level of gene flow between populations A and B caused a deviation from the relationship expected under an IBD model.

## Discussion

Evolutionary divergence is a continuous and dynamic process in which landscape features may limit gene flow between populations. When local ecological factors select for distinct phenotypes in different and genetically isolated populations, or when genetic drift causes different phenotypic traits to become fixed, this process may lead to the development of reproductive isolation (speciation) between populations. Our data sets are the first documentation of high levels of both genetic isolation and phenotypic divergence owing to fine-scale philopatry in cichlids in a high energy fluviatile setting. In both *T. depressum *and *L. tigripictilis *we detected genetic isolation at small geographic scales. In *L. tigripictilis*, we also detected measurable phenotypic differentiation.

In the lower Congo River, both *T. depressum *and *L. tigripictilis *are confined to relatively sheltered shoreline habitats that are in many ways similar to those along the shores of the Great Lakes with a linear array of rocky, sandy, and intermediate substrates. However the hydrodynamics of this powerful river system are strikingly different from those of the Great Lakes. Between the western edge of Pool Malebo and the Atlantic Ocean, the Congo River descends some 280m. This steep drop generates an array of sheltered, isolated habitat patches separated by variously sized, and often extremely powerful, rapids. In the study area, the width of the channel varies more than five-fold, from a maximum of 1.6 km in the Inga Reach to as little as 0.3 km in several intervening narrow chutes. Many of these constricted regions are associated with high-energy rapids that span the entire width of the channel and interrupt stretches of suitable cichlid habitat. Because of these geographic features and the high levels of endemism documented for this river reach, the Lower Congo River represents an important new biohydrologic model system for exploring the isolating mechanisms underlying patterns of species diversification. Consistent with the extreme hydrological forces operating in the LCR, our estimates of genetic isolation are among the highest reported for any African cichlid species on a comparable geographic scale (*cf. *[5,6]).

Two relatively small but intense rapids lie between the most strongly differentiated *T. depressum *populations at Sites A and B. The rapids at Isangala and Fwamalo are both associated with a narrowing of the river channel (0.8 km across at Isangala, 0.3 km at Fwamalo). In both cases, an area of whitewater extends from bank to bank. Analysis of the modest number of available samples at sites B and C did not permit rejection of the null hypothesis of panmixia along the much larger, wider, and shallower rapids at Inga. This pattern may be a result of either reduced statistical power owing to modest sample sizes, or a genuine reflection of population connectivity along the complex shoreline in this stretch with its numerous potential stepping stone habitats.

Perhaps not surprisingly (given its broader distribution and habitat range) the pattern among *Lamprologus tigripictilis *samples is more complex. Assignment tests support the presence of two distinct genetic clusters at Sites A and B, however the probability of belonging to either cluster is not related to the collection locality, and many individuals have intermediate assignment probabilities. A single individual collected from Site C also belongs to one of these two clusters. Almost all individuals at sites D and E assign to a single genetic cluster. Estimates of ϕ_st _between each pair of adjacent collection localities were all significantly different from zero, although we treat this allele frequency based result cautiously because sample sizes were modest, particularly at sites D and E. The highest ϕ_st _estimate was between samples from Sites B and D (the single individual genotype from Site C was excluded from this analysis). This genetic break was also detected in the mtDNA data set. Our morphometric analysis detected phenotypic divergence across this genetic barrier as well, suggesting that the barrier is strong enough for selection or drift to maintain phenotypic differences between populations.

Because the rugged terrain of the LCR did not permit us to collect samples along a formal transect, we cannot unambiguously determine which specific biogeographic barriers maintain population isolation. However the overall patterns of genetic divergence suggest that two species may not respond identically to the same barriers. In *T. depressum*, the strongest genetic break is between Sites A and B, and the two sets of intervening narrow rapids likely play a role in isolating these populations. *Lamprologus tigripictilis *populations from these two sites are also significantly different from each other as measured using ϕ_st_, however the level of genetic differentiation is lower than in *T. depressum *from the same sites or from *L. tigripictilis *populations from further downstream.

In *L. tigripictilis *there is an intriguing general trend of lower genetic diversity at downstream sites relative to upstream sites. However, we detected no evidence of recent genetic bottlenecks in any of the populations, suggesting that the populations have existed in their current state long enough to reach migration-mutation-drift equilibrium. Relative to population B, population D has a large number of private alleles. Indeed, at 8 of the 10 loci, the most common allele in population D was not found in population B suggesting almost no recent gene flow between sites, consistent with long term isolation of these populations. The large number of mtDNA substitutions that distinguish upstream and downstream *L. tigripictilis *haplotypes further supports the idea that the populations have been isolated long enough to have achieved equilibrium.

## Conclusions

The hotspots of species endemism of some African rivers may be less speciose and far less famous than those of the Great Lakes, yet they represent a substantial fraction of cichlid biodiversity and available habitat. It is likely that the observed relationship between landscape forces and population genetic processes observed here is comparable to those operating during the early stages of ecological divergence of many recognized endemic species in the LCR. They may even help illuminate the origins of the spectacular radiations within the Great Lakes. It has been suggested patterns of cichlid evolution and adaptation to high energy river environments may have had an influence on the spectacular patterns of philopatry and evolutionary divergence currently observed in rock-dwelling species in the African Great Lakes [22,23]. Current phylogenenetic analyses indicate that these particular LCR cichlids are not the direct ancestors of the Great Lakes species flocks. However, the complex climate history of Eastern Africa with frequent xeric periods suggests that some of the founders of the Great Lakes faunas would have spent part of their evolutionary history in river systems [24,25]. We hypothesize that philopatry in high energy river systems may be adaptive for these small physoclistous species. If early lake colonists were also philopatric, this 'preadaptation' would have had a profound influence on evolution of species diversity in the African Great Lakes.

## Methods

### Field

*Teleogramma depressum *and *Lamprologus tigripictilis *were collected at multiple sites along a 100 km stretch of the Congo River between the settlements of Kinganga and Matadi (Figure 1). This region includes the Inga Reach, the world's largest and most powerful series of rapids with an elevational drop of 102 m over the 28 km run. Sample sizes and coordinates are shown in Table 1.

Sampling in these habitats is extremely challenging due to a combination of limited river access and extreme hydrological conditions. Nonetheless samples were available from three distinct localities for *T. depressum *(spanning the known distributional range of the species) and five localities for *L. tigripictilis*. Tissue samples were field preserved in 95% ethanol, and voucher specimens were preserved in formalin and transferred to 70% ethanol for morphological analysis. Ethanol preserved tissue samples were stored at -80˚ C until DNA extraction. Collections and specimen processing was conducted in accordance with the American Fisheries Society's Guidelines for The Use of Fishes in Research [26].

### Morphometrics

A set of homologous landmarks [27] was selected to best capture shape variation in each species (additional file 1). Overall shape variation was best described in the dorso-ventrally depressed *Teleogramma *by selecting landmarks from a stable dorsal projection, while in *Lamprologus *variation was captured in lateral projection (Figures 2-A and 2-D). The landmark configuration for each specimen was captured using Tps Dig v2.05 [28] translated, scaled to unit centroid size and rotated with Generalized Procrustes Analysis (GPA). After applying GPA, residual disparity among configurations depends entirely on shape variation and consensus configurations or mean shapes are obtained. Shape variables were analyzed with partial warp analysis (corresponding to a principal components analysis of variance, α = 0), using PCAGen6 [29]. The partial warps summarize the original variance of subsamples/populations and describe major trends in shape change. Hotelling's T statistics were calculated using TwoGroups6h [25] to test for phenotypic differentiation between populations from Sites A-C and D-E for *Lamprologus*, and Sites A and B-C for *Teleogramma*. These partitions were selected based on observed genetic groupings.

### Molecular Techniques

DNA was extracted using Quiagen DNeasy kits according to the manufacturer's instructions.

Mitochondrial DNA haplotypes were determined by sequencing, using published primers, a combined 2044 base pairs comprised of partial cytochrome *b *(Concheiro Pérez *et al*., 2007) and ND2 (Kocher *et al.*, 1995). PCR reactions were performed with Ready-To-Go PCR beads (GE Healthcare) using a thermal profile of 94°C:30; 49-52°C 1:00 and 72°C 1:30 for 30 cycles. PCR products were cleaned with AMPure (Agencourt) prior to a sequencing reaction with Big Dye Terminator Reaction Mix (Applied Biosystems). Sequencing reactions were purified with CleanSEQ (Agencourt), suspended in 0.5 mM EDTA, and run on an ABI 3730xl automated sequencer (Applied Biosystems). DNA sequences used in this study have GenBank accession numbers from HM101344 to HM101461.

A panel of microsatellite loci developed for other cichlid species was screened using DNA from both species, and 10 polymorphic loci were identified for each species. Primer sequences and the GenBank source loci are shown in additional file 2. Primers were purchased with fluorescent dye labels conforming to Applied Biosystems (Foster City, CA, USA) chemistry. All loci were PCR amplified using Applied Biosystems AmpliTaq Gold 2x PCR master mix and a thermal profile of 95°C 1:00; 52°C 1:00 and 72°C 2:00 for 30 cycles.

PCR products were diluted ~1:20 in HPLC grade water and mixed with Applied Biosystems HiDi formamide and GeneScan-600 size standard according to the manufacturer's instructions. Samples were sized on a 3130 xl Genetic Analyzer and fragments were scored using GeneMarker software (SoftGenetics, LLC, State College, PA, USA).

### Analyses

As noted previously collecting fish in many sections of the LCR is logistically challenging and limits the number of individuals available for genetic analysis. Fortunately, the individually based assignment methods implemented in Structure 2.2 do not require *a priori *assumptions of population structure or group membership, and inferences derived using this approach do not rely on precise estimates of allele frequencies within habitat patches [30]. Structure 2.2 was used to determine both the number of likely genetic groups and the affinity of individuals for these clusters. For each species, the likelihood of genetic clusters ranging from 1 to 6 was evaluated using a burn-in time of 10,000 followed by 30,000 repetitions. Simulations were replicated 15 times for each potential number of clusters, and the likelihood scores were averaged to determine the most probable number of clusters. Basic diversity and connectivity statistics were calculated using either GenoDive 2.0b14 [31] and GenAlEx 6.2 [32]. We tested for recent population constrictions using Bottleneck 2.2 [33]. Isolation by distance tests were performed where possible using IBD v 1.52 [34].

Interpopulation mtDNA distances were calculated using MEGA 4.0.2 [35]. Haplotype diagrams were derived from the mtDNA sequences using TCS v1.21 (Clement et al., 2000).

## Authors' contributions

All three authors contributed to the writing of this paper and overall study design. Specimen vouchers and tissue samples were collected by MLJS and RCS during several expeditions to the study region in Bas Congo, Democratic Republic of Congo. JAM initiated the study, and conducted the nDNA microsatellite analyses. MLJS coordinated the project, undertook the morphological analyses and prepared the figures. RCS conducted the mtDNA analyses and prepared the haplotype diagrams. All three authors have read and approved the final version of this manuscript.

## Supplementary Material

Additional file 1 **S-1 **Locations of morphological landmarks Click here for file

Additional file 2 **S-2 **Microsatellite loci and primers used in these analyses. Click here for file
