# Supplementary material for: Genetic isolation and morphological divergence mediated by high-energy rapids in two cichlid genera from the lower Congo rapids
Source: BMC Evol Biol. 2010 May 19;10:149. doi: 10.1186/1471-2148-10-149 (PMC2886069; doi:10.1186/1471-2148-10-149)
Supplement: Additional file 1 — S-1 Locations of morphological landmarks [file 1471-2148-10-149-S1.DOC]

**S-1 Markert, Schelly & Stiassny**

**Definition of landmarks used in morphometric analyses**

Lamprologus

Landmark 1 – premaxillary symphysis

Landmark 2 – median point of hypural flexion

Landmark 3 – posterodorsal margin opercle

Landmark 4 – anterior dorsal spine insertion

Landmark 5 – posterior dorsal ray insertion

Landmark 6 – anterior anal spine insertion

Landmark 7 – posterior anal ray insertion

Landmark 8 – pectoral spine insertion

Landmark 9 – body wall perpendicular to landmark 8

Landmark 10 – body wall perpendicular to landmark 6

Landmark 11 – lateral line perpendicular to landmark 7

Landmark 12 – posterior margin of orbit

Landmark 13 – anterior margin of orbit

Landmark 14 – end of ascending process of premaxillae

Landmark 15 – posterior margin of maxilla

Landmark 16 – anteroventral lachrymal notch

Landmark 17 – angle of preopercle

Landmark 18 – dorsal pectoral ray insertion

Landmark 19 – ventral pectoral ray insertion

Teleogramma

Landmark 1 – anteromedial margin of upper lip

Landmark 2 – anterior dorsal spine insertion

Landmark 3 – right body wall perpendicular to tip of 5^th^ dorsal spine

Landmark 4 – left body wall perpendicular to tip of 5^th^ dorsal spine

Landmark 5 – anterior pectoral ray insertion, right side

Landmark 6 – anterior pectoral ray insertion, left side

Landmark 7 – preopercular sensory canal opening, right side

Landmark 8 – preopercular sensory canal opening, left side

Landmark 9 – mediodorsal margin of orbit, right side

Landmark 10 – mediodorsal margin of orbit, left side

Landmark 11 – right nares

Landmark 12 – left nares

Landmark 13 – posterior margin of orbit

Landmark 14 – anterior margin of orbit

Landmark 15 – anteromedial margin of lachrymal fold

Landmark 16 – posterior extent of upper lip fold, right side

Landmark 17 – posterior extent of upper lip fold, left side

Landmark 18 – dorsal point of hypural flexion
